# Plastamination, Human Health, and Countries’ Cultural Orientation: An Exploratory Study on Prevention Strategies and Organizational Policies and Practices

**DOI:** 10.3390/ijerph23030382

**Published:** 2026-03-17

**Authors:** Giuseppe Perna, Rosaria Meccariello, Luisa Varriale

**Affiliations:** 1 Department of Business Management, Pegaso University, 80143 Naples, Italy; giuseppe.perna@unipegaso.it; 2 Department of Medical, Human Movement, and Well-Being Sciences, University of Naples Parthenope, 80133 Naples, Italy; 3 Department of Economics, Law, Cybersecurity and Sports Sciences, University of Naples Parthenope, 80035 Nola, Italy; luisa.varriale@uniparthenope.it

**Keywords:** plastamination, human health, technology, microplastics, nanoplastics, ecosystem, biodiversity, cultural factors, organizational policies, bibliometric analysis

## Abstract

**Highlights:**

**Public health relevance—How does this work relate to a public health issue?**

- According to the World Health Organization, *“health risks exist at all stages of the plastic lifecycle, from production and use to recycling and disposal, as well as from legacy plastics in the environment”*.
- Plastic contamination (plastamination) and exposure to plastic debris in the micro and nano-range by ingestion, dermal contact or inhalation is inevitable, and the related health risks concern its intrinsic toxicity, accumulation in biological tissue and function as a vector for other contaminants like heavy metals, plasticizers and so on.

**Public health significance—Why is this work of significance to public health?**

- This study examines plastamination through a broad and integrated approach, combining biological, medical, engineering, managerial and cultural perspectives, in order to offer a systematic and multidimensional reading of the phenomenon.
- This approach will be useful for the definition of strategies and policies for the mitigation of plastamination and the preservation of both environment and human health.

**Public health implications—What are the key implications or messages for practitioners, policy makers and/or researchers in public health?**

- The study offers an innovative contribution to the understanding of plastamination and to the definition of multidimensional intervention strategies and policies to address one of the most pressing challenges of our time.
- This study provides interesting suggestions about the possible influence of cultural and social contexts on effective organizational and policy solutions to foster plastamination also supported by technological innovation.

**Abstract:**

In recent decades, the increasingly widespread diffusion of plastic contamination (plastamination) has attracted growing attention from both the scientific and public community due to its harmful effects on human health and environmental balance. Several studies have highlighted a link between exposure to microplastics and nanoplastics and the onset of central nervous system problems, impaired reproductive function, altered liver metabolism, dysbiosis and other chronic diseases. At the same time, research has highlighted how plastamination negatively impacts natural ecosystems, accelerating biodiversity loss and compromising the stability of the planet. Faced with these critical issues, scientific and professional debate has progressively shifted toward defining effective and sustainable strategies, often based on innovative technologies, aimed at limiting the overall impact of this global phenomenon. This study, consisting of a systematic literature review through a bibliometric analysis in the Web of Science database (1974–June 2025), aims to examine plastamination through a broad and integrated approach, combining biological, medical, engineering, managerial and cultural perspectives, to offer a systematic and multidimensional reading of the problem. Preliminary findings underscore the importance of an integrated vision that brings together technology, culture, society, and medicine, emphasizing the need for shared global policies and collective responsibility. The study thus aims to offer an innovative contribution to the understanding of plastamination and to the definition of multidimensional intervention strategies to address one of the most pressing challenges of our time.

## 1. Introduction

In recent decades, plastic production has seen exponential growth, becoming one of the most widely used resources in numerous sectors, from medical and healthcare to technology and food [1]. This diffusion is closely linked to plastic’s intrinsic characteristics: lightness, versatility, corrosion resistance, and low production costs. However, these very qualities, which have favored its widespread use globally, have contributed to one of the most serious environmental emergencies of our time [2]. The average lifespan of plastic products is approximately four years, but their persistence in the environment can extend for centuries. Consequently, a minimal portion is recycled or incinerated, while the majority accumulates in landfills or in the environment, progressively fragmenting into microplastics, particles smaller than 5 mm in diameter [3].

Plastic contamination (here referred to as plastamination) represents one of the most urgent and complex issues today, as these particles, originating both from the degradation of plastic waste and from primary sources such as microbeads contained in consumer products, have now reached a global level of pervasiveness. They have been identified in various environmental matrices and even in food, with direct implications for human health [1,4,5]. Exposure therefore appears inevitable, and the related risks concern both the intrinsic toxicity of plastic and its function as a vector for other contaminants: heavy metals, antibiotics, pathogenic microorganisms, and plasticizers [5]. This phenomenon not only amplifies the consequences for public health, but also introduces new critical issues related to food safety and the food chain, where bioaccumulation phenomena are observed.

The scientific literature over the past six decades has documented the effects of microplastics on the human body. However, despite research progress, numerous questions remain unanswered, especially regarding the effects of chronic exposure under realistic conditions. Experimental studies are still limited, and understanding the mechanisms of interaction between microplastics and living organisms requires further multidisciplinary effort.

At the same time, the consequences for natural ecosystems are becoming increasingly evident. Environmental plastamination compromises biodiversity and threatens the survival of many species. In this sense, plastamination cannot be considered a purely technical or environmental problem, but rather a complex phenomenon that intertwines biological, medical, social, and cultural aspects. Its management requires not only the adoption of advanced technologies but also the development of organizational policies, educational programs, and integrated preventive practices. Recent studies highlight a persistent research gap in the literature concerning the investigation of plastic contamination as a complex and multidisciplinary phenomenon, in which behavioral, social, and cultural dimensions have been examined [6,7,8,9,10], alongside proposals and analyses of plastic-related policies and educational training programs [11,12].

From this perspective, our exploratory study aims to analyze the phenomenon of plastamination by adopting a markedly multidisciplinary approach, which not only allows us to understand its scientific and environmental dynamics but also to identify effective organizational practices and initiatives to prevent it. The problem of microplastics cannot be addressed solely from a technical or health perspective, but requires the development of management, educational, and training programs that actively involve civil society, institutions, and various economic actors. Specifically, this study, through a bibliometric analysis of the literature on plastamination from 1974 to June 2025, aimed at identifying key emerging trends, outlining possible future developments, and identifying effective organizational solutions for the prevention and management of the phenomenon. The analysis adopts a multidisciplinary perspective and recognizes the central role of cultural and social factors, highlighting how plastamination represents a global challenge that requires the integration of scientific knowledge, technological innovation, and collective responsibility.

Considering the stated objectives and the study’s bibliometric and multidisciplinary approach, the research is guided by the following questions:

RQ1: What are the main thematic strands and emerging trends and disciplinary perspectives in the literature on plastamination and microplastic contamination (1974–June 2025)?

RQ2: What future-oriented directions can be anticipated based on the evolution of publications, citation patterns, and terminological co-occurrences identified through bibliometric analysis?

RQ3: Do prevention strategies and organizational and policy solutions mentioned in the literature appear most promising for facing plastamination?

Indeed, countries’ cultural orientation is a crucial factor in defining prevention strategies, as the methods of production, consumption, and management of plastic waste vary significantly depending on social and geographical contexts. In this sense, the fight against plastamination is not limited to a scientific issue, but rather a global challenge that must combine technical knowledge, collective responsibility, and adaptation to cultural specificities. The paper is structured as follows: the “Plastamination: Origins and Evolution of Microplastic Contamination” Section analyzes the origins and historical development of the phenomenon of plastic contamination, reconstructing its main stages and evolutionary dynamics. Section 2 and Section 3 present the research methodology and results of the qualitative study, respectively. Section 4 offers a discussion of the findings, while Section 5 concludes with key insights, research limitations, and future directions.

### Plastamination: Origins and Evolution of Microplastic Contamination

Today, plastic is a ubiquitous material; this is primarily due to its high annual production [13], its chemical and physical characteristics (such as ductility and resistance), its low production costs, and its use in a wide variety of fields (medical, textile, agricultural, industrial, and food) [14]. Plastic is a synthetic organic material, formed from the polymerization of individual organic molecules [15,16]. This polymer is forcefully entering our everyday lives, simplifying common daily tasks and leading to our current “modern” lifestyle.

Although plastic has positively revolutionized our reality since the 1950s [17], its excessive use and incorrect disposal have made it a real threat, as well as a global environmental and health emergency. Indeed, plastic materials are often not properly recycled or sent to landfill but can be released into the environment accidentally or intentionally [13,18,19]. Once in the environment, plastic becomes an environmental contaminant, causing not only visual pollution but also a problem for marine organisms, as it interacts mechanically with them and can be ingested, leading to gastrointestinal tract obstruction [20,21]. Furthermore, the permanence of plastic waste in the environment causes the deterioration of its mechanical and physicochemical properties, leading to the degradation of the material and the formation of plastic fragments, which are considered microplastics when their size is <5 mm [22]. These small particles can further degrade into nanoparticles [23], be ingested by organisms and release intrinsic contaminants (plasticizers) or environmental contaminants, adsorbing to their surfaces [24,25,26]. This is because plastic persists in the environment for a very long period, which is still not well understood, and can even last hundreds to thousands of years in some cases [18,19,27,28,29]. Since most plastics are not biodegradable materials, one of the problems associated with plastamination is its ubiquity, i.e., its presence in all environments. It can circulate in the sea thanks to currents and be distributed in all environmental matrices (water, sediment, organisms) thanks to the processes of flotation, deposition, and ingestion.

Although larger pieces of waste attract the most attention, there is a growing awareness that smaller and seemingly insignificant plastic fragments are even more harmful and dangerous [30], effectively posing a huge problem for ecosystems, both marine and continental [31]. Microplastics, as mentioned, are plastic particles smaller than 5 mm. In addition to their polymeric and structural composition [13], they can be divided into different categories based on their shape: sphere or pellet, fragment, film, and fiber are the most common and recognized by the scientific community. In particular, the terms sphere, pellet, and microbead all refer to pseudospherical plastics, which differ in their origin and use. The term fragment is often used to describe rigid, thick particles of irregular shape. Since microplastics already exist in this form, such as abrasives used in cosmetics, the more accurate term for these would be “irregular particles.” Films, on the other hand, are planar microplastics in which one of the three dimensions is significantly smaller than the other two. Plastics with a length/diameter ratio >3 are instead described as “fibers” or “filaments” [32].

Their presence in the environment can be traced back to various sources: they can be found in cosmetics, personal care and household products, and construction and industrial products, or they can form following the use and disposal of plastic products. Depending on their origin, microplastics can be divided into two main groups [33]: (i) Primary microplastics include particles and fibers produced as such, which can be released into the environment accidentally or during the use of products containing them, such as toothpastes and exfoliants, hand and facial soaps, and cosmetics [34], and are transported through wastewater, eventually dispersing into the seas and oceans. These particles generally appear flattened, cylindrical, spheroidal, or disk-shaped. (ii) Secondary microplastics are formed from the fragmentation of larger plastics, under the influence of various atmospheric agents (sunlight, precipitation, salt water, wave motion, etc.) and other environmental factors [35]. In this case, the microplastics produced have fragments with irregular morphology, ranging from angular to rounded, depending on the degree of wear. In addition to fragments, one of the most common types in this class is fibers, which appear as thin, elongated filaments. Recent studies have shown that this type is the most common in marine environments, with percentages exceeding 70–80% in particle number per m^3^ [36]. A massive presence of fibers in the environment is linked to high discharges through the sewer system following the washing of synthetic garments [37].

Because they persist in the environment for a long time [31], microplastics can be ingested by organisms living in marine and coastal environments at various trophic levels and thus enter the food chain [21], causing harm at all levels of the food web, including to humans [38,39]. Once ingested by organisms, microplastics can cause direct physical harm: they bypass biological barriers and are absorbed by biotic tissue, organs, and even cells, exhibiting acute and (sub)chronic toxicity, carcinogenicity, and developmental and reproductive toxicity [31]. Further risks may arise from the feeling of satiety experienced by organisms caused by the ingested particles; they will tend to eat less, resulting in decreased growth [28,31,40].

Moreover, plastic fibers have recently been found in drinking water, which could carry toxic substances potentially hazardous to human health [31]. Indeed, given their high specific surface area, micro- and nanoplastics can adsorb persistent, bioaccumulative, and toxic (PBT) hydrophobic chemicals and trace metals present in the surrounding seawater, acting as contaminant vectors [28,29,41]. Among all plastic waste, therefore, micro- and nanoplastics are of particular concern in terms of potential negative effects on the environment and on human and animal health, as they act as vectors for a variety of invasive and harmful species worldwide. The key issue with pollutants is concentration; currently, significant concentrations have been reached, resulting in negative effects on wildlife but, fortunately, not yet (or at least not visibly) on humans. Since these materials remain in the environment once they reach it, as they cannot be decomposed, a change is needed now that concentrations do not yet appear to be worrying; otherwise, we risk reaching a tipping point beyond which regression will no longer be possible. The prevalence of microplastics in marine and terrestrial environments poses a significant risk to our food security: the WWF (Worldwide Fund for Nature) has estimated that we ingest more than 5 g of microplastics every week, the equivalent of a credit card. The ability of these particles to absorb environmental pollutants exposes humans to a veritable mix of harmful chemicals [42]. In this context, the Emerging Microplastic Syndrome (EMS) hypothesis offers a potential key to interpreting the systemic effects of chronic exposure to microplastics. If these particles are not only physical foreign bodies but also carriers of chemical contaminants and heavy metals, their biological impact could be amplified by the combination of mechanical action, toxicological burden, and the body’s inflammatory response. The recent theoretical model proposed by Umberto Cornelli and colleagues [43] suggests that the accumulation and persistence of micro- and nanoplastics in tissues may contribute to the onset of a chronic, low-grade inflammatory state, potentially aggravated by the presence of persistent, bioaccumulative, and toxic substances adsorbed on their surfaces. In this sense, the ability of microplastics to act as carriers of contaminants strengthens the hypothesis of a systemic health risk, transforming an environmental problem into a potential global public health issue.

Taken together, plastic debris in the micro- and nano-range enters the food chain and is capable of transporting different environmental toxicants; nevertheless, plastic debris also includes emerging airborne pollutants that can migrate through various environmental pathways. In this respect, plastic debris poses a potential threat to human health due to its ability to enter the body not only through ingestion, but also through inhalation and dermal contact, to cross biological barriers, and to accumulate in biological tissues [5], thus contributing to the onset of diseases. Table 1 summarizes the main clinical evidence reporting the presence of plastics in the micro- and nano-range within the human body in diseases.

## 2. Materials and Methods

In reviewing the main contributions in the literature, the primary focus is divided into three main objectives: (1) to identify and examine new and emerging trends in the field of plastamination considering several scientific disciplinary perspectives (biology, medicine, management, engineering, and so forth), (2) to predict future-forward trends in plastamination, and (3) to identify and propose effective organizational solutions (practices, policies, actions, programs and so on) to prevent and manage the plastamination phenomenon, starting from cultural and social values and considering the role of technologies as well as of the entire global community.

Within the framework of this systematic literature review (SLR), it is essential to establish a precise terminological distinction between two concepts that, despite their phonetic resemblance, refer to fundamentally different phenomena and belong to entirely separate scientific domains. As already outlined, the term “plastamination” or, more broadly, plastic contamination, refers to the presence, accumulation, and ecological or biological impact of plastic-derived materials (including microplastics, nanoplastics, and macroplastic debris) in environmental matrices such as water bodies, soils, sediments, and living organisms. This concept is firmly grounded in environmental science, ecotoxicology, and public health research, and constitutes the central subject of inquiry in the present review.

We therefore conducted a systematic literature review to map and evaluate the existing body of literature, identify knowledge gaps, and delineate the limitations of the current state of knowledge on the examined topic. The systematic review is predicated on a dynamic interplay between the analyzed sources and interpretative analysis. Specifically, systematic review methodology aims to minimize bias and synthesize findings as objectively as possible. This methodological approach was adopted because it enables the coherent organization of a structured body of information, facilitates the assessment of critical dimensions of the phenomenon under investigation, and permits the examination of multiple research questions. Furthermore, this method is effective in identifying areas of uncertainty requiring clarification and underexplored domains of inquiry, thereby stimulating the development of subsequent research. To conduct systematic reviews of the current research literature, VOSviewer software (1.6.16) was employed for mapping and analyzing thematic trends, author and institutional collaborations, and the geographical distribution and temporal evolution of research [57,58].

The study identification process was conducted with an advanced search within the digital library Web of Science, a database that provides an overview of academic literature in the fields of science, technology, medicine, social sciences, arts, and humanities. For this selection, the following search criteria were used: the presence of the keywords “microplastics,” “technology,” and “human health” in the title and abstract of published works, combined using the Boolean operators (AND, OR). In our SLR we combined topic terms explicitly anchored to plastamination (microplastic *, nanoplastic *, plastic debris, plastic pollution, plastic contamination, plastic fragment *, plastic particle *, macroplastic *, microbeads, plastic fiber *) with environmental scope qualifiers (environment *, ecosystem *, marine, aquatic, freshwater, terrestrial, soil, sediment *, biota, food chain, human health, ecotox *), and included explicit exclusion strings to filter out plastination-related content. Furthermore, the search strategy incorporated explicit Boolean exclusion terms targeting plastination-related literature (e.g., NOT “plastination” OR “anatomical preservation” OR “tissue preservation” OR “polymer impregnation” OR “von Hagens”). Geographical restrictions were not used to retrieve as many documents as possible, but a time limit was applied, considering only documents from 1974 to June 2025 to locate recent information and data. Subsequently, all duplicates and articles irrelevant to the review were excluded, and other relevant searches were added. The final selection criteria were articles published in a journal that adopts a peer-reviewed review process, as well as studies containing qualitative or quantitative research and focusing on the factors that may influence the management of a resource with a disability within a corporate work context.

We therefore developed a database in which contributions from the literature, predominantly of an international nature, were classified according to specific analytical dimensions, including journal of publication, with particular attention to identifying the most authoritative outlets in terms of impact factor, year of publication, total citation count, average annual citation rate, study type (distinguishing between theoretical and empirical approaches), and methodological framework employed. Literature mapping was subsequently performed using VOSviewer software; specifically, the software’s term identification function was utilized to systematically identify the principal themes addressed in the analyzed articles. A minimum occurrence threshold of 10 was established, such that a term was required to appear in the title and/or abstract of at least 10 distinct articles to be included among the candidates for mapping. The adoption of this threshold ensures a robust representation of inter-term relationships within the map and prevents the inclusion of insignificant or erroneous terms [57,58]. The choice of a minimum occurrence threshold of 10 was motivated by the need for methodological robustness and statistical significance. In a term co-occurrence analysis, too low a threshold would result in the inclusion of marginal or sporadic concepts, generating information noise and reducing the clarity of the thematic map; conversely, an excessively high threshold would risk excluding emerging but already established topics in the scientific debate. The threshold of 10 occurrences therefore represents a balance between inclusiveness and selectivity, ensuring that each included term is sufficiently representative within the analyzed corpus. This choice ensures the stability of the co-occurrence relationships calculated through the association strength measure and improves the reliability of the generated clusters, avoiding biases resulting from rare or structurally irrelevant terms. To prepare the terms for the mapping phase, VOSviewer calculates the correlation between terms present in the selected articles through a measure based on association strength. Specifically, the degree of correlation between terms in the map is determined by the co-occurrence ratio between two terms relative to the strength of association that each term exhibits with others within the analyzed corpus. This procedure resulted in the identification of 29 recurring terms in the selected articles, together with their respective frequencies, defined as the number of occurrences of each term in the overall corpus. Furthermore, a relevance score was calculated for each of the 29 terms, and based on this score, the most significant terms were selected. The identification of exactly 29 terms does not result from an arbitrary selection, but from the rigorous application of the parameters set in the analysis software. Once the minimum threshold of 10 occurrences was defined and the automatic calculation of co-occurrences through the measurement of association strength was applied, VOSviewer returned a set of terms that simultaneously satisfied the criteria of frequency and statistical relevance. The final number of 29 terms therefore represents the objective result of the intersection between (i) the minimum required frequency, (ii) the relevance score calculated by the software, and (iii) the normalization of the relationships between terms in the selected corpus. In other words, 29 represents the number of concepts that, based on the methodological parameters adopted, possess a significant presence and structurally solid relationships within the analyzed dataset. The final number of articles analyzed is therefore the result of a progressive selection process based on clearly defined key parameters. First, the search string, applied to the title and abstract in the Web of Science database, generated an initial set of records. Second, duplicates and documents irrelevant to the research objectives were removed. Third, inclusion criteria were applied regarding publication type (peer-reviewed articles only), methodological nature (qualitative or quantitative studies), and thematic consistency with the treatment of the phenomenon under analysis. Further exclusions were made following a thorough reading of abstracts and full texts, where necessary. The final number of articles considered therefore represents the result of the combination of initial search criteria, time filters, scientific quality criteria, and content relevance assessment, ensuring methodological consistency, process transparency, and study replicability. Subsequently, the VOSviewer clustering algorithm was applied, which is predicated on an optimization process designed to systematically identify groups of terms, or thematic clusters, based on their interrelationships. The algorithm maximizes the sum of association strengths between pairs of terms belonging to the same cluster while minimizing overall cluster size [57,58]. To identify the clusters, the default VOSviewer resolution value of 1.0 was employed, and a minimum cluster size of two terms was established, which was deemed sufficient for analysis and graphical representation. Through this procedure, the VOSviewer clustering algorithm identified six distinct thematic clusters.

## 3. Results

### 3.1. Bibliometric Analysis

**Figure 1:**
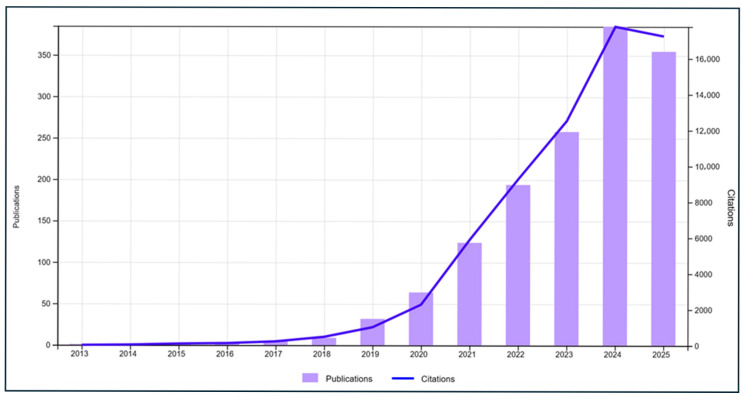
Times cited and publications over time.

Over the past six decades, the increasing production and diffusion of plastic have led to environmental contamination on a global scale, with significant implications for human health and ecosystems. This bibliometric analysis examines the evolution of research on plastic contamination, identifying key trends, emerging technologies, and knowledge gaps, with a focus on organizational, cultural, social, and medical dimensions. The bibliometric approach highlighted not only the volume and trends of scientific production, but also knowledge gaps and emerging areas where advanced technologies can make a significant contribution. Overall, the search returned to 1438 items, including articles, abstracts, etc., with an h-index of 123 and an average citation rate per year of 46.62; these are, for the most part, theoretical studies that adopt a qualitative methodology. Figure 1 shows significant research activity over the last five years, with the greatest production in 2024, and a trend in citations that suggests continued interest in the topic, especially since the 1990s. Awareness of the problem of microplastic pollution first emerged in 1993, when global plastic production increased from 2.1 million tons in 1950 to 147 million tons in 1993. In the mid-1990s, companies began incorporating microparticles into cosmetics and cleaning products, but it was only with the advent of the third millennium that people began to talk about microplastics and the alarming consequences of their spread in our oceans [59].

**Figure 2:**
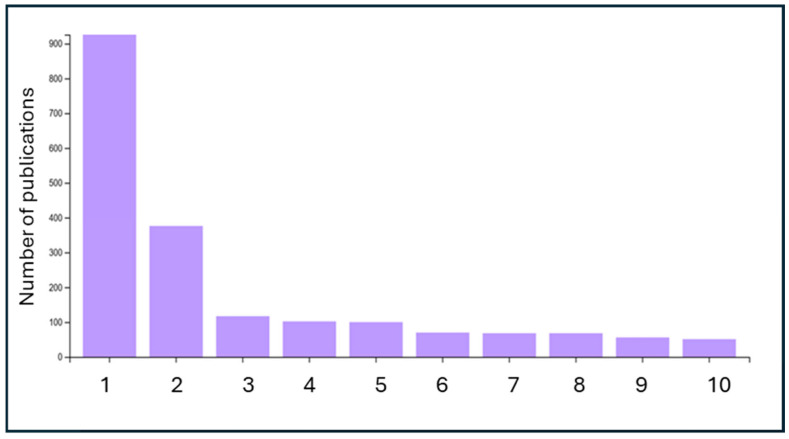
Scientific area to which publications belong: 1. environmental sciences; 2. environmental engineering; 3. toxicology; 4. chemical engineering; 5. water resources; 6. marine freshwater biodiversity; 7. multidisciplinary chemistry; 8. public environmental health; 9. multidisciplinary materials science; and 10. nanoscience and nanotechnology.

Publications can then be grouped into different scientific research areas. Figure 2 shows that the scientific area related to environmental sciences has the highest number of publications, at 926.

Most publications on microplastics fall within the environmental sciences because the phenomenon was initially discovered and studied in seas, rivers, and soils—that is, in natural contexts. Microplastics behave like genuine environmental pollutants, subject to dispersion, accumulation, and interaction with complex ecosystems. The main questions—where they are found, how they move, and what effects they have on organisms—typically belong to environmental ecology and chemistry. Although the research involves diverse disciplines (chemistry, medicine, engineering), environmental sciences have served as the center of gravity because they provide the methodological tools and the scientific language with which the problem has been described. Furthermore, the support of public bodies and NGOs, along with the media representation of microplastics as a global ecological problem, has consolidated this disciplinary affiliation.

**Figure 3:**
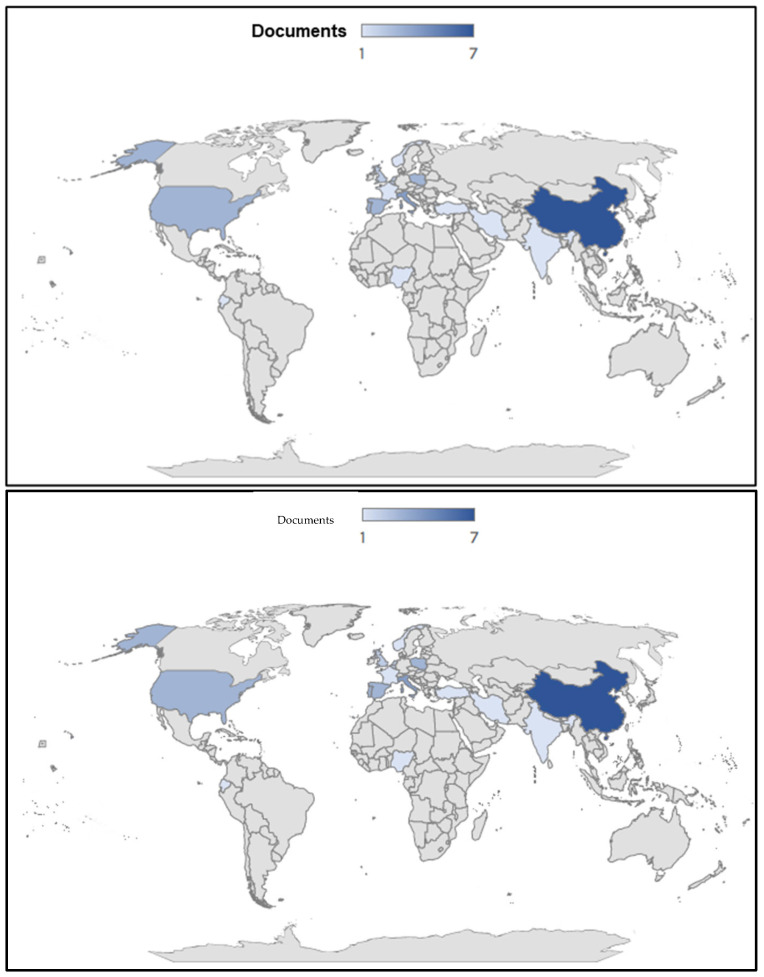
Geographical distribution of publications.

Figure 3 highlights how the topic has been analyzed across numerous areas worldwide, as microplastic pollution is a global problem and one of the most pressing environmental emergencies of modern times, requiring advanced scientific knowledge and timely monitoring systems to be fully understood and effectively addressed. China (17.5%) represents the country with the highest percentage of relevant publications, followed by Italy (10%), the United States (7.5%), Portugal (7.5%), Spain (7.5%), and Poland (7.5%). Indeed, as confirmed by the current situation in China, many Chinese companies are among the world’s leading producers of single-use plastic. In 2015 alone, 367,000 tons of plastic waste were dumped into the Yangtze River (the longest in China and Asia), making it the waterway with the largest amount of such materials globally [60]. In 2019, according to data provided by the European Plastics Association, 51% of total production came from Asia. China alone accounted for over 32% of global plastics, more than double the 15% produced by Europe and exceeding the 19% held by Canada, Mexico, and the United States combined. In the 2022 update, the situation appears substantially unchanged: China remains in first place (32%), followed by North America (18%), Europe (15%), other Asian countries (17%), Africa and the Middle East (8%), Latin America (4%), and Japan (3%). It is therefore not surprising that approximately 86% of the plastic discharged into the sea comes from Asian rivers, while 7.8% comes from African rivers [60]; however, it should be noted that these regions have recently increased their efforts related to waste management, including plastic waste, especially after the COVID-19 pandemic. Until 2017, Beijing was the world’s leading destination for the export of plastic waste, playing a key role in the treatment of plastic, one of the most complex materials to recycle. However, in early 2018, Chinese authorities introduced an import ban on various types of waste, including plastic [61]. Despite this, China has made significant progress in recent years in environmental protection, placing the reduction in plastic use among the central objectives of its green agenda. In 2022, for example, the People’s Republic released the first research report on the fight against plastic pollution, confirming its global leadership in recycling and reuse practices. To understand the government’s approach, it is essential to consider the project called “Replace Plastic with Bamboo”: a three-year national plan that aims to gradually eliminate non-biodegradable single-use plastics in urban and rural contexts, replacing them with bamboo products to reduce contamination [62]. Bamboo is a highly renewable natural resource: reaching maturity in three to five years, a well-managed forest can be harvested and exploited repeatedly for decades, up to 40–70 years. Furthermore, unlike plastic, bamboo products are completely biodegradable and do not end up in landfills. Although not all Asian governments show the same level of commitment, these data together paint a very different picture than the commonly held one.

### 3.2. Two-Dimensional Map

To visualize in detail the relationships between terms and thematic trends (i.e., “Network Visualization”; “Density Visualization”; “Overlay Visualization”), we constructed a two-dimensional map using VOSviewer [57,58].

**Figure 4:**
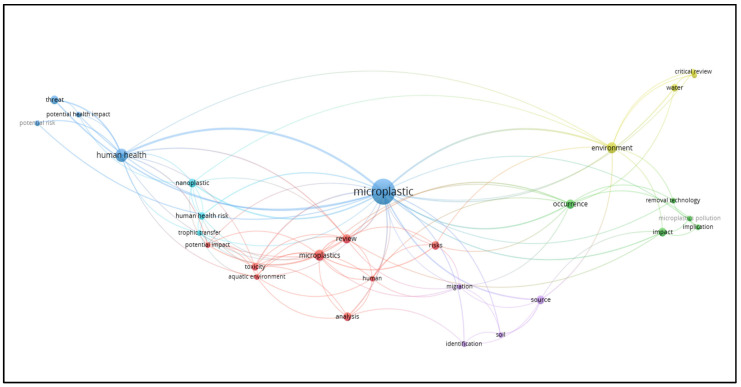
Network Visualization. Cluster 1, red: studies on the chemistry of plastics; Cluster 2, green: studies exploring innovative technologies; Cluster 3, blue: studies on the medical use of plastics; Cluster 4, yellow: studies evaluating the effects of plastic polymers on environmental safety and chemical waste management; Cluster 5, purple: studies on the cultural interest concerning plastamination for scientific dissemination, educational exhibitions, and public awareness; Cluster 6, light blue: studies highlighting the importance of integrating plastamination into the organizational protocols of laboratories and educational institutions.

The Network Visualization (Figure 4) displays the six thematic clusters based on their associated color:

- Cluster 1 (red): Most of the work focuses on the chemical and mechanical techniques of plastamination, including the development of advanced polymers.
- Cluster 2 (green): Recent studies explore the use of innovative technologies such as microfluidics, three-dimensional imaging, and nanotechnologies.
- Cluster 3 (blue): Some articles discuss the use of plastic for medical devices and its potential impact on human health.
- Cluster 4 (yellow): Some studies have begun to evaluate the effects of polymers used on environmental safety and chemical waste management. This cluster, which encompasses issues related to the dimension of sustainability and environmental impact, is still emerging, indicating a gap in the literature on the combination of technological advancement and ecological responsibility.
- Cluster 5 (purple): Several studies show that cultural interest concerns the use of plastamination for scientific dissemination, educational exhibitions, and public awareness. Furthermore, the literature suggests how social perception of plastamination can influence the acceptance of advanced technologies, and how scientific communication is essential to avoid misunderstandings or cultural resistance.
- Cluster 6 (light blue): A growing number of studies highlight the importance of integrating plastamination into the organizational protocols of laboratories and educational institutions. The need for operational guidelines, risk management, staff training, and policies to minimize environmental and chemical impacts is also emphasized.

In summary, given the exponential growth of the relevant literature, with an estimated 90% of indexed publications having appeared after 2015, the present work acknowledges an inherent temporal asymmetry in the retrieved corpus. Early foundational works (1974–2003), largely indexed under alternative terminology (e.g., ‘plastic debris’, ‘plastic litter’, ‘marine plastic pollution’), were specifically targeted through supplementary backward citation searches and manual screening of seminal reference lists, in order to ensure historical representativeness of the evidence base.

**Figure 5:**
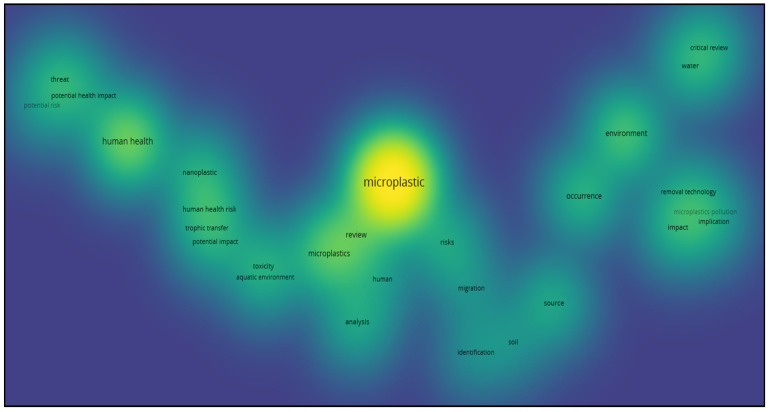
Density Visualization. Colors ranging from yellow to blue indicate, based on their intensity, the number of articles associated with topics within a given space on the map.

In the Density Visualization (Figure 5), colors ranging from yellow to blue indicate, based on their intensity, the number of articles associated with topics within a given space on the map. Specifically, yellow indicates areas with comparatively high search intensity, while blue indicates thematic areas with comparatively lower search intensity. The literature review was conducted to analyze both the technical aspects of plastamination and its organizational, cultural, social, and medical dimensions. Keyword analysis allowed us to identify connections between research fields and highlight the multidisciplinary nature of plastamination. It emerged that while technical keywords dominate the literature, the organizational, social, and medical dimensions are even less developed, representing a significant area of opportunity for further research.

**Figure 6:**
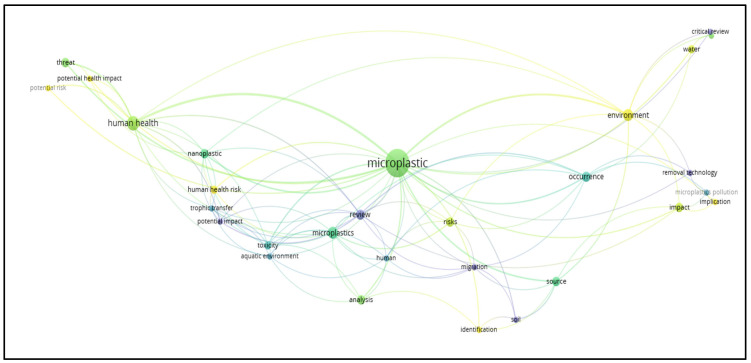
Overlay Visualization. Recent publications are in bright yellow; older articles are represented with cooler colors such as blue.

Finally, with Overlay Visualization (Figure 6), we can identify terms present in articles that belong to more recently published research; specifically, the latter are represented in the map with a bright yellow color, while topics present in research published in older articles are represented with cooler colors such as blue. The result of this analysis is consistent with what is shown in Figure 1, which emphasized that research activity on the topic has been concentrated in the last five years. The analysis shows that studies on plastamination initially appeared sporadically in the first decades (1960s–1980s), mainly as pioneering works on possible health implication and production trends. The scientific literature of this period focused mainly on technical aspects, with a focus on the use and safety of plastics in medical devices or drug interaction with medical plastics. Since the 1990s, the volume of publications has increased steadily, alongside the introduction of advanced technologies, such as specialized silicone polymers, epoxy resins, and microinjection systems to improve the quality and durability of plastics. Over the past two decades, the literature has expanded to include analyses of human health monitoring, clinical applications, and anatomical laboratory management, indicating a growing awareness of the need to integrate technology with organizational practices and safety protocols.

Bibliometric analysis thus highlights growing scientific attention to plastic contamination and the importance of advanced technologies in its management. However, to effectively address this problem, an integrated approach is needed that also considers organizational, cultural, social, and medical dimensions. Collaboration between researchers, institutions, and communities is crucial to developing sustainable solutions and promoting a significant change in production and consumption practices.

## 4. Discussion

Our systematic review of the literature has highlighted that plastamination cannot be considered exclusively a technical issue: its effective application requires a systemic, multidisciplinary, and preventive approach, capable of integrating technology, organization, culture, society, and medicine. In recent decades, and particularly in the last twenty years, the issue of plastic and its spread into the environment has emerged with such force that it has become one of the major global emergencies [5]. Plastic, once celebrated as a symbol of progress, modernity, and technological innovation, is now the subject of growing concern due to the negative effects it produces on a global scale. Initially associated with undeniable advantages—being lightweight, resistant, economical, and versatile—this class of materials has progressively transformed into a disruptive element of environmental balance and, indirectly, human health. The ubiquitous spread of plastic waste, now traceable in every corner of the planet, from the depths of the oceans to the highest layers of the atmosphere, highlights the scale of a phenomenon that can no longer be ignored and requires systemic and global intervention strategies [63].

Reflections on plastamination are not just about waste management: they are a problem that encompasses ecological, biological, health, economic, and even ethical dimensions. Indeed, plastic does not just occupy physical spaces in ecosystems, but interacts with natural cycles, penetrates living organisms, bypasses biological barriers, bio-accumulates in tissues, alters biological functions, and produces imbalances whose effects are not yet fully predictable [5,64,65]. In this sense, it represents a paradigm of contemporary challenges: a material created to improve daily life has transformed, through indiscriminate use and inadequate management, into a widespread and pervasive threat.

A particularly sensitive issue concerns the effects of plastic on human health. Until relatively recently, public debate focused primarily on the visible impact of plastic waste, such as islands of plastic in the oceans or animals trapped in nets and bags. Only in more recent years has scientific research begun to systematically investigate the less obvious, but potentially more serious, consequences associated with the presence of microplastics and nanoplastics in the human body. These tiny fragments, resulting from the degradation of larger plastic products, are now found in a wide range of environmental and food matrices: in drinking water, seafood, table salt, beer, honey, and even the air we breathe [66]. The routes of exposure, therefore, are multiple and include inhalation, ingestion, and, to a lesser but not negligible extent, dermal absorption. Once in the body, these particles are not easily eliminated and tend to accumulate in tissues and organs, giving rise to inflammatory processes and possible alterations in biological functions [67]. One of the few studies that assessed the average overall exposure to microplastics estimated the amount of microplastics ingested per capita at 39,000–52,000 particles per year, the majority of which comes from the atmospheric dust we breathe, while the main food sources are seafood and bottled water [68]. The effects of microplastics on humans have been studied recently [69] but to a limited extent due to ethical constraints, strict safety measures for handling human samples, and still poorly developed microplastic detection techniques. Initially, studies aimed to understand the effects of micro- and nanoplastic particles released from medical implants [70] and the effects of certain plastic additives. More recently, studies have begun on microplastics ingested through food and airborne dust and their ability to cross biological barrier with consequences on neurological, cardiovascular, digestive, respiratory and reproductive functions (see Table 1 for clinical data in human). At the same time, it has been observed that several chemical additives used in the production of plastics, such as phthalates and bisphenol A, have endocrine-disrupting effects, compromising hormonal function and fertility. These substances, capable of mimicking or inhibiting the action of natural hormones, are associated with reproductive problems, metabolic alterations, and an increased risk of hormone-dependent tumors and neurological and developmental disorders [71,72,73,74].

The effects of plastic contamination extend far beyond the human body and involve the entire terrestrial ecological system. The oceans are where the problem is most evident: tons of plastic waste reach the seas every year, transported by rivers, uncontrolled landfills, and urban drainage systems. These materials, resistant to natural degradation, persist for decades or centuries, progressively fragmenting into ever smaller particles [5]. Marine fauna is significantly affected by this process. Fish, mollusks, crustaceans, turtles, and seabirds ingest microplastics, mistaking them for food. Ingestion can lead to suffocation, intestinal obstruction, dysbiosis, malnutrition, and death [75]. Even when not immediately lethal, microplastics can compromise an organism’s the ability to feed, reproduce, and defend themselves from predators, thus reducing the resilience of animal populations. No less serious are the indirect effects, linked to bioaccumulation and biomagnification: toxic substances adhering to microplastics can travel up the food chain to reach humans, accentuating the link between environmental pollution and human health [71,72,76]. But plastic is not just a marine problem. The atmosphere itself is not exempt: microscopic plastic fibers released from synthetic fabrics, tires, and other sources are carried by the wind and fall everywhere, reaching even remote regions, such as Alpine glaciers or the Arctic [77]. The widespread accumulation of plastic in ecosystems results in a progressive deterioration of fundamental ecosystem services, such as climate regulation, water purification, soil fertility, and biodiversity. This scenario threatens the stability of natural environments and, consequently, the very survival of the Earth and its living species.

Another crucial aspect concerns the role of technology and organizational practices in preventing the negative effects of plastic contamination. A first line of action involves the development of advanced recycling technologies. Despite progress, the actual recycling rate of plastic remains relatively low at the global level. Many types of plastic are not easily recyclable or require expensive and energy-intensive processes.

However, new techniques, such as chemical recycling, promise to overcome some of these limitations by transforming plastic waste into secondary raw materials [34]. Practical examples in this area are multiplying: in Europe, projects like Plastic Energy are using pyrolysis to convert mixed plastic waste into oils, reusable by the petrochemical industry; in Japan, several companies have developed pilot plants that transform plastic into liquid fuels for energy production. At the same time, interest in bioplastics and biodegradable materials is growing. These polymers, derived from renewable sources such as starch, cellulose, or algae, have the potential to reduce waste accumulation, but still have limitations related to resistance, cost, and actual biodegradability under natural environmental conditions [78]. Some large-scale retail chains have already begun introducing biodegradable corn starch-based packaging for fruit and vegetables, while in the biomedical field, disposable devices made from PLA (polylactic acid) are being tested as a replacement for traditional plastic. Nevertheless, these bio-plastics are surely free for the environment, but their effects on biological systems are poorly understood, and preliminary data in the field are not promising [79]. Another perspective concerns the application of nanotechnologies and advanced filtration systems, aimed at removing microplastics from water and air. These are promising solutions but still in the experimental phase, requiring further studies to evaluate their sustainability and scalability. For example, research groups in Germany are testing nanomaterial-based filter membranes for urban wastewater treatment plants, while US startups have designed filter devices for washing machines capable of intercepting synthetic microfibers released from fabrics during washing [80]. Finally, a central element is the circular economy, which proposes a paradigm shift: no longer linear production based on extraction, consumption, and disposal, but a virtuous cycle that involves reuse, recycling, and the valorization of materials. Some cities, such as Amsterdam and Milan, have already implemented door-to-door waste collection systems that achieve levels of plastic recovery much higher than the European average; textile companies, such as Patagonia, produce technical clothing from recycled PET bottles, transforming waste into a resource [81]. While scientific innovations allow the development of advanced tools for environmental monitoring, such as sensors capable of detecting the presence of microplastics in water and air, or artificial intelligence platforms capable of analyzing enormous amounts of data to identify the main sources of dispersion, there is also a need to integrate these resources into appropriate organizational models. Management practices, both at the industrial and institutional levels, play a key role: more efficient waste management plans, traceability systems throughout the entire product life cycle, environmental certifications, and extended producer responsibility policies are examples of strategies that help prevent the release of plastic into ecosystems. In this sense, the experience of Norway, where plastic bottle producers are required to finance a deposit system with recovery rates of over 95%, represents a concrete model of application [82]. It is essential to encourage businesses to adopt environmentally and socially sustainable practices by ensuring that people acquire relevant information and awareness about sustainable development through the promotion of eco-sustainable using through eco-labels, environmental claims, or environmental product declarations. Technology, therefore, should not be understood only as a tool for ex-post “repair,” but as a preventative lever capable of reducing risks at the source. In this sense, the integration of technological innovation and sustainable organizational models represents the essential condition for addressing the problem structurally, while simultaneously ensuring environmental protection, the safeguarding of human health, and economic competitiveness.

Beyond technological solutions, the role of public policies and collective awareness is essential. Without stringent regulation and a cultural shift in everyday behavior, efforts risk remaining ineffective. Measures such as the ban on single-use plastics, recycling incentives, more stringent environmental standards, and extended producer responsibility systems are essential tools for reducing the production and release of plastic into the environment [81]. Civil society, for its part, must develop a critical awareness of plastic use, adopting more sustainable and responsible consumption practices. Awareness-raising campaigns, environmental education, and the promotion of sober lifestyles are fundamental tools for guiding individual behavior toward a lower environmental impact. The analysis of plastamination cannot ignore the recognition of the role that cultural dimensions and national differences play in the perception, management, and prevention of this phenomenon. Plastic pollution is not, in fact, a purely technical process, reducible to parameters of chemical toxicity or measurable ecological impacts; on the contrary, it is intimately linked to social practices, consumption patterns, symbolic values, and collective norms that vary significantly from one context to another. In this sense, culture becomes an interpretative filter through which individuals and communities define what is risky, what is acceptable, and what strategies should be adopted to mitigate environmental damage. The sociological and anthropological literature has extensively demonstrated that the perception of risk is never a purely scientific fact, but rather a social construct. Mary Douglas and Aaron Wildavsky, with their cultural theory of risk [83], have highlighted how societies tend to develop representations of dangers based on moral and organizational values. Translated to the field of plastamination, this means that not all countries react in the same way to the growing scientific evidence on the harmful effects of microplastics and nanoplastics: some adopt stringent policies and launch widespread educational campaigns, while others struggle to consider plastic as a priority problem compared to other economic and social emergencies [83].

The diversity of national responses is the product of multiple factors. First, shared value systems and beliefs profoundly influence how people conceive of their relationship with nature. In societies with a strong individualistic tradition, typical of many Western countries, consumption is often considered a fundamental right, and restrictive policies that limit the use of single-use plastics encounter resistance in the name of personal freedom. Conversely, in collectivist contexts, such as in many Asian cultures, there is a greater willingness to accept common rules if they are perceived as serving the good of the community. This distinction, although simplified, helps us understand why similar regulations can have divergent outcomes depending on the cultural fabric in which they are introduced.

No less important is the influence of religious and spiritual traditions, which can be a valuable resource for promoting environmentally friendly practices, but can also sometimes be an obstacle if rigidly anchored to customs that involve the use of plastic materials. For example, in India, the sacredness attributed to rivers makes the phenomenon of water pollution by plastic particularly alarming, encouraging social and religious mobilizations against irresponsible waste disposal. In Japan, the culture of order, harmony, and collective cleanliness has fostered a sophisticated waste sorting and recycling infrastructure, which has found widespread acceptance among the population. In contrast, in many Western societies, environmental mobilization has often been expressed through social movements and non-governmental organizations, which have pushed institutions to regulate single-use plastics not so much for religious or moral reasons, but rather out of a secular sense of responsibility towards future generations [84].

Culture is also manifested in the most banal daily practices, such as eating habits. In many countries, the development of street food consumption or the spread of fast food has led to an increase in the use of single-use plastic packaging, with direct consequences on waste production. In other contexts, however, the tradition of reusing and conserving objects has limited the impact of these practices. It is therefore not just a question of regulations or technology, but of actual lifestyles that shape the spread of plasticization. New generations, exposed to global media campaigns, tend to demonstrate greater environmental awareness, but this does not imply homogeneity in behavior: the practical translation of this awareness varies greatly depending on economic possibilities, available infrastructure, and social expectations [85].

The role of environmental education appears crucial to understanding national differences. Countries such as Germany, Sweden, and Japan have introduced sustainability-focused education programs for decades, resulting in citizens demonstrating greater competence and motivation to adopt environmentally friendly practices. Conversely, in contexts where the school system does not adequately integrate the environmental dimension, the population tends to underestimate the risks associated with plastic, prioritizing immediate convenience over long-term sustainability. Education is not just about schools, but also universities, the media, and social networks, which convey cultural representations that can strengthen or weaken collective ecological awareness. It is well known that viral social media campaigns have played a decisive role in reducing the use of plastic straws in several Western countries: a prime example of how digital culture can accelerate behavioral changes on a global scale.

Added to these elements is the institutional and political dimension. Public policies are never implemented in a cultural vacuum, but rather are set within a context of trust or distrust in institutions. In Northern European countries, characterized by high levels of institutional trust, environmental regulations generally meet with greater adhesion and respect. In other contexts, where corruption or the lack of credibility of authorities are perceived as endemic problems, even the most ambitious policies risk being ignored by the population. Consequently, the effectiveness of anti-plastamination strategies depends not only on regulatory content, but also on the cultural legitimacy that institutions manage to build.

In conclusion, environmental plastamination represents a current challenge that requires immediate and coordinated responses. The impacts on human health, particularly on the nervous system and reproduction, along with the devastating consequences for ecosystems, demonstrate how complex and interconnected the problem is. However, thanks to advances in scientific research, technological innovation, and a growing environmental awareness, it is possible to imagine a future in which plastic, no longer a threat to life on the planet, becomes part of a more sustainable production system that respects the natural balance.

## 5. Concluding Remarks, Limitations, and Future Directions

Plastamination represents one of the most serious paradoxes of modernity: the material that has improved the quality of life in so many sectors is proving to be a widespread threat to ecosystems and human health. The phenomenon’s origin lies in the linear economic model based on disposable goods, while its evolution follows the dynamics of globalization and mass production. This study confirms that effectively addressing this problem requires an integrated approach, combining scientific research, technological innovation, international regulations, and changes in individual behavior. Only in this way will it be possible to reduce the impact of microplastics and preserve the health of the planet and future generations.

The present study offers several original contributions to the emerging, interdisciplinary field of plastamination research. By conducting a systematic bibliometric analysis of the scientific literature spanning the period of 1974–2025, this work provides a comprehensive and empirically grounded mapping of the intellectual landscape surrounding plastic contamination and its implications for human health, organizational governance, and cultural contexts. The analysis documents a significant and accelerating growth in scholarly output on plastamination, confirming both the centrality of advanced technologies in the management of polymeric materials and the increasingly cross-disciplinary nature of the field. A key original contribution of this study lies in its integration of dimensions that remain structurally underrepresented in the predominantly technical literature: namely, the organizational, managerial, cultural, and national dimensions of plastamination governance. By examining plastamination through the intersecting lenses of biological science, medical research, engineering, organizational theory, and cross-cultural analysis, this study proposes a multidimensional interpretive framework that transcends sectoral approaches. In particular, the analysis highlights that a country’s capacity to develop effective responses to plastamination depends not only on available economic resources and technological infrastructures, but critically on the degree of coherence between public policies, educational systems, and shared cultural models. This finding reinforces the need for intercultural dialog and context-sensitive strategies in the design of global sustainability interventions. Furthermore, the application of conceptual frameworks such as the TOE (Technology–Organization–Environment) model and multi-helix innovation models—including the quadruple and quintuple helix—represents an innovative analytical contribution, offering structured tools for aligning technological innovation, organizational capacity, and environmental governance in addressing the systemic challenge of microplastic contamination.

Despite the breadth of the existing scientific production on plastamination, the bibliometric analysis conducted in this study reveals several structural gaps that currently limit the translation of research findings into effective practices and policies. First, the integration of advanced technologies within public organizations, research centers, and applied laboratories remains fragmented. While numerous contributions describe technically promising solutions for the detection, removal, or management of plastic contaminants, comparatively few explore the conditions under which such solutions can be implemented in real-world institutional settings, with adequate attention to safety protocols, traceability requirements, and waste management criteria. This disconnection between technological innovation and organizational implementation reflects the persistence of rigid disciplinary boundaries and insufficient investment in technology transfer processes and personnel training. Second, the cultural and social dimensions of plastamination remain markedly underappreciated in the current literature. Existing studies are predominantly focused on chemical and biological measurements, with limited attention devoted to the dynamics of science communication, public risk perception, and the social acceptance of proposed environmental interventions. This gap is significant insofar as the social legitimacy of environmental technologies and policies depends as much on the robustness of empirical data as on their comprehension and interpretation by citizens, professionals, and decision-makers. The absence of research systematically exploring cultural narratives, information processes, and the behavioral determinants of adoption substantially limits the capacity to design effective and widely accepted interventions at regional and national scales. Third, on the medical and prevention front, the literature presents promising but fragmented results. Understanding of the toxicological profiles of different polymers and additive compounds, as well as the effects of co-exposure to contaminants transported by microplastics, remains incomplete. Systematic studies on occupational protection protocols for healthcare and laboratory professionals exposed to microplastics during diagnostic or training procedures are particularly scarce. Finally, the environmental sustainability of plastamination-related interventions is insufficiently addressed. Few studies comprehensively assess the life cycle of the materials employed, their recyclability, or the potential ecological impact of process-derived waste, creating the risk that technically effective interventions may generate unintended collateral environmental impacts.

From an organizational perspective, it is essential to develop safe and efficient operational protocols capable of regulating the use, management, and disposal of plastic materials, with the aim of minimizing risks to health and the environment. Adequate organization, supported by shared standards and monitoring systems, is an essential prerequisite for ensuring not only the effectiveness of interventions but also their replicability in different contexts. The medical dimension, in turn, draws attention to the potential effects of plastamination on human health, particularly for professionals and students involved in research, experimentation, or training activities. Protecting health through preventive measures, safety protocols, and awareness programs is a priority, as exposure to microplastics and their derivatives poses risks that are still partly unknown and require further scientific investigation. Furthermore, the environmental dimension is at the heart of a broader reflection on sustainability. Promoting environmentally responsible practices and reducing the impact of polymers on the ecosystem is not only a matter of environmental protection, but also an essential step to ensure the continuity of natural balances and the protection of future generations. From this perspective, plastamination becomes a platform for testing innovative integrated management strategies capable of combining technological progress, social well-being, and environmental responsibility. Finally, analyzing plastamination through the lens of cultural and national differences highlights the intrinsically complex and multidimensional nature of the phenomenon. Plastamination, far from being a purely technical or scientific problem, is profoundly intertwined with the values, traditions, religious beliefs, social norms, and daily practices that characterize different geographical contexts [86]. A country’s ability to develop effective responses to plastamination depends not only on economic resources or available technologies, but above all on the degree of coherence between public policies, educational systems, and shared cultural models. This highlights the need for a truly interdisciplinary approach, capable of integrating ecological, health, political, and anthropological dimensions, recognizing that the fight against plastamination can only be based on intercultural dialog and differentiated strategies capable of respecting the specificity of local contexts while pursuing a global sustainability objective.

Despite the breadth of scientific production on plastamination, bibliometric analysis highlights structural gaps that limit its effectiveness when translated into practices and policies. First, the integration of advanced technologies within public organizations, research centers, and application laboratories remains fragmented: many contributions describe promising technical solutions, but few explore how these solutions can be implemented in real-world settings while respecting safety, traceability, and waste management criteria. This disconnect between innovation and organizational management is partly due to the persistence of rigid disciplinary boundaries and insufficient attention to technology transfer processes and the training of personnel who will operate the new tools. This creates the risk that potentially useful technologies remain confined to the experimental stage or, worse, are adopted without adequate protocols, increasing rather than mitigating the risks associated with handling polymeric materials. The existing limitations of our study may serve as starting points for future research. For instance, future studies could systematically examine and classify all health-related claims according to the level of evidence supporting them, distinguishing explicitly between: findings from in vitro experimental studies; findings from in vivo animal models; epidemiological and observational evidence in human populations; and speculative or hypothesis-driven statements. Claims regarding neurological effects (including associations with Alzheimer’s and Parkinson’s disease), reproductive toxicity, and blood–brain barrier penetration should be presented with appropriate epistemic qualification, maintaining a clear and consistent distinction between correlation, mechanistic plausibility, and established causal evidence throughout. In this direction, we may identify and analyze possible and effective organizational policies and practices to prevent and manage plastamination with a specific focus on its impact on human health.

At the same time, the cultural and social dimensions of plastamination appear underappreciated in the current literature. Studies focus primarily on chemical and biological measurements, paying less attention to the dynamics of scientific communication, public perception of risks, and social acceptance of the proposed practices. This gap is significant because the social legitimacy of environmental technologies and policies depends as much on the robustness of data as on their understanding and perception by citizens, professionals, and decision-makers. The lack of research exploring information processes, cultural narratives, and the factors that influence behavioral adoption reduces the ability to design effective and shared interventions on the regional scale. On the medical and prevention front, the literature shows promising but fragmented results. Understanding the toxicity of different polymers, additive compounds, and the effects of co-exposure to contaminants transported by microplastics is still incomplete; similarly, systematic studies on protection protocols for healthcare and laboratory workers exposed during diagnostic or training procedures are scarce. This picture highlights the need for longitudinal and biological monitoring studies that can inform clinical guidelines and personal protection standards, as well as training initiatives aimed at integrating toxicological skills and biosafety practices. Finally, the environmental sustainability of the solutions used in plastamination deserves greater attention. Few studies comprehensively assess the life cycle of the materials used, their recyclability, or the potential ecological impact of waste resulting from technical processes. Without a systematic environmental analysis comparing the ecological benefits and costs of different technologies, there is a risk of introducing interventions with unwanted collateral impacts. These limitations highlight the need to integrate the bibliometric approach with qualitative studies, empirical investigations, and clinical evaluations to obtain a more comprehensive and applicable view of the implications of plastamination in organizational, cultural, and healthcare contexts.

Considering these critical issues, fertile ground is opened for future research that effectively adopts a multidisciplinary perspective: studies that integrate technology engineering with organizational sciences, social sciences, toxicology, and environmental assessments could fill the identified gaps, enabling the design of effective, safe, and socially acceptable solutions. Only by building collaborative networks between academia, institutions, and stakeholders will it be possible to translate emerging knowledge into practices and policies capable of sustainably addressing the complexity of plastamination. The challenge of microplastics therefore requires a systemic approach, capable of combining scientific research, technological innovation, and social responsibility. Only by combining the skills and tools of multiple disciplines will it be possible to develop effective strategies to reduce the impact of plastamination and ensure the protection of human health and biodiversity.

From this perspective, addressing the issue of microplastics requires the creation of communities of research and practice that involve not only environmental scientists, but also toxicologists, physicians, materials engineers, policy makers, and communication experts. The pervasive nature of the phenomenon, which crosses ecological, social, and health boundaries, highlights the need for a truly interdisciplinary approach. Conceptual frameworks such as the TOE (Technology–Organization–Environment) model can offer useful tools for understanding how to align technological innovations (e.g., filtering systems or new biodegradable materials), organizational capabilities (monitoring and waste management networks), and environmental factors (e.g., ecological and climatic dynamics that amplify the dispersion of particles). In this context, the adoption of a multi-helix innovation model, such as the quadruple or quintuple helix, highlights the urgent need for collaboration between scientific research, industry, public institutions, civil society, and environmental protection, so as to transform the problem of microplastics into an opportunity to rethink the relationship between production, consumption, and sustainability.

The gaps identified above delineate a fertile and urgent agenda for future interdisciplinary research. From a methodological standpoint, future studies should integrate the bibliometric approach adopted here with qualitative investigations, longitudinal empirical studies, and clinical evaluations, to obtain a more comprehensive and practically applicable understanding of the organizational, cultural, and health-related dimensions of plastamination. The development of longitudinal biological monitoring studies and the formulation of clinical guidelines and personal protection standards for occupationally exposed populations represent particularly pressing priorities. Furthermore, future research should devote greater systematic attention to the cultural and national determinants of plastamination governance, exploring how values, social norms, religious beliefs, and institutional frameworks shape both public risk perception and the adoption of preventive behaviors. Comparative cross-national studies, drawing on established frameworks such as Hofstede’s cultural dimensions, could make a significant contribution in this direction. More broadly, effectively addressing the systemic challenge of microplastic contamination will require the construction of collaborative research and practice communities that bring together environmental scientists, toxicologists, physicians, materials engineers, policy makers, and communication experts. Only through such integrated, multi-stakeholder networks—aligned with the logic of quadruple and quintuple helix innovation models—will it be possible to translate emerging scientific knowledge into practices and policies capable of sustainably addressing the full complexity of plastamination, ensuring the protection of human health, biodiversity, and the ecological foundations on which future generations depend.

## Figures and Tables

**Table 1:** Clinical evidence of different plastic debris in human diseases.

Human Biological Sample	Plastic Type	Sample Size	Plastic Size	Clinical Observations	Reference
Blood	PE, PS, PVC, PP, PA66	N = 21 Parkinson’s disease patients N = 12 age- and sex-matched healthy controls	Not specified	Elevated blood levels of PVC, PP and PA66 in Parkinson’s disease patients	[44]
Ileal segments and adipose tissue	PE, PU, ACR, PMMA, PP, PVC, EVA, Fluororubber, CPE, BR, PS, PET	N = 10 Crohn’s disease patients	20.34 to 437.17 μm	MP concentrations correlated positively with the severity of intestinal fibrosis	[45]
Liver	PS, PVC, PET, PMMA, POM, PP	N = 6 with liver cirrhosis N = 5 controls	3–29.5 µm	MPs in individuals with liver cirrhosis, but not in controls	[46]
Decedent human kidney, liver and brain cortex	Mainly PE over 12 different polymers (N66, ABS, SRB, PET, N6, PMMA, PU, PP, PVC, PC, PS)	N = 12 patients with confirmed dementia N = 28 controls (collected in 2016) N = 24 controls (collected in 2024)	1–5 µm	Greater accumulation of MNPs in the cohort of decedent brains with documented dementia diagnosis	[47]
Cerebrospinal fluid	PP, PVC, PE, PS	Cohort 1: N = 17 amyloid-positive subjects N = 15 amyloid-negative subjects Cohort 2: N = 11 amyloid-positive subjects	1 nm ∼ 5 mm	Positive correlation with Alzheimer’s disease Gradual accumulation of microplastics in the cerebrospinal fluid associated with cognitive decline among AD individuals	[48]
	PE, PP, PVC, PS, PC, PA6, PA66	N = 48 intracranial aneurysms patients N = 108 leptomeningeal metastasis patients	MPs and NPs	Higher PP and PVC levels in the cerebrospinal fluid related to increased intracranial aneurysms risk	[49]
Carotid plaques	PE, PVC	N = 257	Smaller than 1 µm	Higher risk of a composite of myocardial infarction, stroke, or death in patients with MPs in carotid artery plaque	[50]
Nasal lavage fluids	Not specified	N = 30 controls N = 36 patients with allergic rhinitis	Not specified	Significantly higher levels of MP density in the nasal lavage of allergic rhinitis group	[51]
Placenta	PE, PS	N = 43	<10 μm	Correlation with reduced fetal growth in intrauterine growth restriction pregnancies	[52]
Placental chorionic villi	PE, PVC, PS, PP	N = 31 participants in their first trimester	NMPs	Positive association between the abondance of MPs and unexplained spontaneous miscarriage	[53]
Ovarian follicular fluid	Not specified	N = 18	<10 µm with mean diameter: 4.48 µm (3.18–5.54 µm)	Significant correlation with Follicle Stimulating Hormone Low correlation with estradiol and Body Mass Index	[54]
Amniotic fluid	PTFE, PS, ABS	N = 48	3.05 ± 1.05 µm	No significant associations between MP exposure and immediate adverse pregnancy outcomes	[55]
Semen and urine	PS, PP, PE, PS, PTFE, PET, PC, ABS	N = 113	1.2–20 μm	MP exposure was associated with a significant decrease in total sperm number, sperm concentration, and progressive motility	[56]

ABS, acrylonitrile butadiene styrene; ACR, crylate copolymer; BR: butadiene rubber; CPE, chlorinated polyethylene; EVA, ethylene–vinyl acetate; MP, microplastic; N6, nylon-6; N66, nylon 66; NP, nanoplastic; PA6, polyamide 6 Nylon 6; PA66, polyamide 66 Nylon 66; PC, polycarbonate; PE, polyethylene; PET, polyethylene terephthalate; PMMA, polymethyl methacrylate; POM, polyoxymethylene; PP, polypropylene; PS, polystyrene; PTFE, polytetrafluoroethylene; PU, polyurethane; PVC, polyvinyl chloride; SRB, styrene–butadiene rubber.

## Data Availability

The original contributions presented in this study are included in the article. Further inquiries can be directed at the corresponding author.
